# Withaferin a Triggers Apoptosis and DNA Damage in Bladder Cancer J82 Cells through Oxidative Stress

**DOI:** 10.3390/antiox10071063

**Published:** 2021-06-30

**Authors:** Tsu-Ming Chien, Kuang-Han Wu, Ya-Ting Chuang, Yun-Chiao Yeh, Hui-Ru Wang, Bi-Wen Yeh, Chia-Hung Yen, Tzu-Jung Yu, Wen-Jeng Wu, Hsueh-Wei Chang

**Affiliations:** 1 Graduate Institute of Clinical Medicine, College of Medicine, Kaohsiung Medical University, Kaohsiung 80708, Taiwan; u108801005@kmu.edu.tw; 2 Department of Urology, School of Medicine, College of Medicine, Kaohsiung Medical University, Kaohsiung 80708, Taiwan; bewen90@yahoo.com.tw; 3 Department of Urology, Kaohsiung Medical University Hospital, Kaohsiung 80708, Taiwan; 4 Graduate Institute of Medicine, College of Medicine, Kaohsiung Medical University, Kaohsiung 80708, Taiwan; u108500037@kmu.edu.tw; 5 Department of Biomedical Science and Environmental Biology, College of Life Science, Kaohsiung Medical University, Kaohsiung 80708, Taiwan; u107023007@gap.kmu.edu.tw (Y.-T.C.); u108551005@gap.kmu.edu.tw (Y.-C.Y.); whr0319@gmail.com (H.-R.W.); 6 Graduate Institute of Natural Products, Kaohsiung Medical University, Kaohsiung 80708, Taiwan; chyen@kmu.edu.tw (C.-H.Y.); u109831002@kmu.edu.tw (T.-J.Y.); 7 Center for Cancer Research, Kaohsiung Medical University, Kaohsiung 80708, Taiwan

**Keywords:** Withaferin A, bladder cancer, DNA damage, apoptosis, oxidative stress

## Abstract

Withaferin A (WFA), the Indian ginseng bioactive compound, exhibits an antiproliferation effect on several kinds of cancer, but it was rarely reported in bladder cancer cells. This study aims to assess the anticancer effect and mechanism of WFA in bladder cancer cells. WFA shows antiproliferation to bladder cancer J82 cells based on the finding of the MTS assay. WFA disturbs cell cycle progression associated with subG1 accumulation in J82 cells. Furthermore, WFA triggers apoptosis as determined by flow cytometry assays using annexin V/7-aminoactinomycin D and pancaspase detection. Western blotting also supports WFA-induced apoptosis by increasing cleavage of caspases 3, 8, and 9 and poly ADP-ribose polymerase. Mechanistically, WFA triggers oxidative stress-association changes, such as the generation of reactive oxygen species and mitochondrial superoxide and diminishment of the mitochondrial membrane potential, in J82 cells. In response to oxidative stresses, mRNA for antioxidant signaling, such as nuclear factor erythroid 2-like 2 (*NFE2L2*), catalase (*CAT*), superoxide dismutase 1 (*SOD1*), thioredoxin (*TXN*), glutathione-disulfide reductase (*GSR*), quinone dehydrogenase 1 (*NQO1*), and heme oxygenase 1 (*HMOX1*), are overexpressed in J82 cells. In addition, WFA causes DNA strand breaks and oxidative DNA damages. Moreover, the ROS scavenger *N*-acetylcysteine reverts all tested WFA-modulating effects. In conclusion, WFA possesses anti-bladder cancer effects by inducing antiproliferation, apoptosis, and DNA damage in an oxidative stress-dependent manner.

## 1. Introduction

Urothelial carcinoma is the most common cancer in the urinary system, including the urinary bladder and upper urinary tract. Most urothelial carcinoma occurs in the urinary bladder, annually showing an estimated 550,000 new cases and 200,000 deaths. Most urinary bladder urothelial carcinoma is classified as non-muscle-involved bladder cancer (NMIBC), showing a high five-year survival of 96% for patients diagnosed early. Still, this survival dramatically decreases to 6% when tumor metastasis occurs [1]. However, these bladder cancer patients’ mortality is high in advanced diseases, even surgery [2]. Therefore, novel drug development in bladder cancer therapy is warranted.

Several natural products and chemicals provide reactive oxygen species (ROS) modulation and induce apoptosis of cancer cells, which is valuable for anticancer treatment [3,4,5,6,7,8]. For example, Withaferin A (WFA) is a cell-permeable [9] steroidal lactone derivative from *Withania somnifera*, a traditional Indian Ayurvedic medicine used for centuries. WFA is a well-known antioxidant, anti-inflammatory [10], and anticancer [11,12,13,14,15,16,17] natural product with an ROS modulating ability [12,18,19], but its antiproliferation function and mechanism in bladder cancer cells remains unclear.

In the present study, we investigated whether WFA exhibits an antiproliferation effect on bladder cancer cells. The urinary transitional cell carcinoma J82 cell line was used in vitro for evaluating antiproliferation and underlying WFA anticancer mechanisms in apoptosis, oxidative stress, antioxidant, and DNA damage systems. In addition, pretreatment with the ROS inhibitor *N*-acetylcysteine (NAC) elucidated the ROS modulating role of WFA. Therefore, this study sheds light on the antiproliferation function and mechanism of WFA in bladder cancer treatment.

## 2. Materials and Methods

### 2.1. Cell Lines, Drugs, and Survival Assay

The human urinary bladder urothelial carcinoma cell line (J82) was procured from the American Type Culture Collection (ATCC, Manassas, VA, USA). Cell culture conditions were Dulbecco’s modified Eagle’s medium containing 10% fetal bovine serum in a 5% CO_2_ atmosphere of a standard incubator.

WFA and apoptosis inhibitor Z-VAD-FMK (ZVAD) [20] were obtained from Selleckchem.com (Houston, TX, USA) and dissolved in dimethyl sulfoxide (DMSO). The antioxidant NAC (Sigma-Aldrich; St. Louis, MO, USA) was used to test the function of oxidative stress in WFA-induced changes. Cell survival was measured by the tetrazolium-based MTS kit (Promega Corporation, Madison, WI, USA) [21].

### 2.2. Cell Cycle Analysis

Following 75% ethanol fixation, the DNA content of drug-treated cells was detected by staining with 7-aminoactinmycin D (7AAD) (1 μg/mL, 30 min) (Biotium; Hayward, CA, USA) [22] for Accuri C6 flow cytometry (Becton-Dickinson, Mansfield, MA, USA).

### 2.3. Apoptotic Annexin V/7AAD and Pancaspase Assays

The Annexin V-stained and pancaspase-detected apoptotic cells were analyzed using the annexin V/7AAD kit [23] (Strong Biotech; Taipei, Taiwan) and generic caspase (caspases-1 and 3 to 9) detecting kit [21] (Abcam, Cambridge, UK) as described by the user’s manual for Accuri C6 flow cytometry.

### 2.4. Apoptotic Western Blotting Assay

The apoptosis sampler antibodies, including cleaved poly (ADP-ribose) polymerase (c-PARP), cleaved caspases-3 (c-Cas 3), c-Cas 9, and c-Cas 8, were used in 1:1000 dilution (Cell Signaling Technology, Inc., Danvers, MA, USA). In addition, a loading control mAb-β-actin antibody was purchased from Sigma-Aldrich (St. Louis, MO, USA). Other procedures for Western blotting were previously described [20].

### 2.5. Cytometric ROS, Mitochondrial Superoxide (MitoSOX), and Mitochondrial Membrane Potential (MMP) Assays

The ROS, MitoSOX, and MMP contents of drug-treated cells were detected by using 2′,7′-dichlorodihydrofluorescein diacetate (DCFH-DA; Sigma-Aldrich) [24] (10 μM, 30 min), MitoSOX™ Red [25] (50 nM, 30 min), and DiOC_2_(3) [26] (Invitrogen; San Diego, CA, USA) (5 nM, 30 min), respectively. The contents were detected by Accuri C6 flow cytometry.

### 2.6. Real-Time PCR for Antioxidant Pathway Genes

Total RNA was extracted and reverse-transcribed by using Trizol solution (Invitrogen) and an OmniScript RT kit (Qiagen, Valencia, CA, USA) [27]. The touch-down program [28] was performed for real-time PCR detection of the antioxidant genes [29,30], such as nuclear factor erythroid 2-like 2 (*NFE2L2; NRF2*), catalase (*CAT*), superoxide dismutase 1 (*SOD1*), thioredoxin (*TXN*), glutathione-disulfide reductase (*GSR*), quinone dehydrogenase 1 (*NQO1*), and heme oxygenase 1 (*HMOX1*), as previously mentioned [31]. The fold activation (log2) of antioxidant mRNA expression was calculated by the 2^−ΔΔCt^ method [32] in reference to the *GAPDH* gene.

### 2.7. Cytometric γH2AX and 8-Hydroxy-2-Deoxyguanosine (8-OHdG) Assays

Following 75% ethanol fixation, the γH2AX [4] and 8-OHdG [33] contents of drug-treated cells were detected using Accuri C6 flow cytometry as previously described. Briefly, the γH2AX antibody [4] (Santa Cruz Biotechnology; Santa Cruz, CA, USA) (4 °C, 1 h) coupled with Alexa Fluor^®^488-conjugated secondary antibody (Cell Signaling Technology) and 7AAD incubation (5 μg/mL, 30 min) were supplied to fixed cells. For 8-OHdG detection, fixed cells were provided with an 8-OHdG-FITC antibody (Santa Cruz Biotechnology) (4 °C, 1 h).

### 2.8. Statistical Analysis

The significance in multiple comparisons was determined by one-way analysis of variance (ANOVA) accompanied by Tukey HSD post hoc examination. Data labeled with different lower-case letters indicate significant differences.

## 3. Results

### 3.1. WFA Inhibits Proliferation of Bladder Cancer Cells

**Figure 1:**
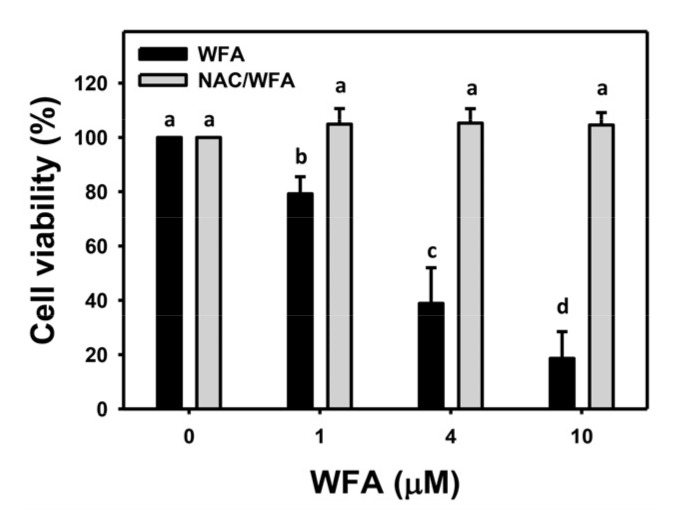
Cell viabilities of WFA in bladder cancer cells. The ROS inhibitor *N*-acetylcysteine (NAC) pretreated effect on the cell viability (24 h MTS assay) of bladder cancer cells (J82) following WFA incubation was also detected. Cells were pretreated with NAC (8 mM, 1 h) and post treated with WFA (0 to 10 μM for 24 h), where the negative control for WFA (0 μM) contained 0.1% DMSO. Data, mean ± SD (*n* = 3). Columns showing non-overlapping lower-case letters indicate *p* < 0.05 for multiple comparisons. For example, WFA for 0, 1, 4, and 10 μM treatments shows “a, b, c, d”, indicating significant differences among each other because they do not overlap with the same lower-case letters. Similarly, WFA 1 μM and NAC/WFA 1 μM showing “a” and “b” indicate significant differences among each other.

WFA reduces cell viability (%) of bladder cancer J82 cells in dose-dependent manners (Figure 1). Pretreatment with the oxidative stress inhibitor NAC was performed to elucidate the dependence of oxidative stress on the antiproliferation function for WFA. WFA-induced antiproliferation in J82 cells at different concentrations was recovered to the normal proliferation condition by NAC (Figure 1).

### 3.2. WFA Accumulates SubG1 and G2/M Populations in Bladder Cancer Cells

**Figure 2:**
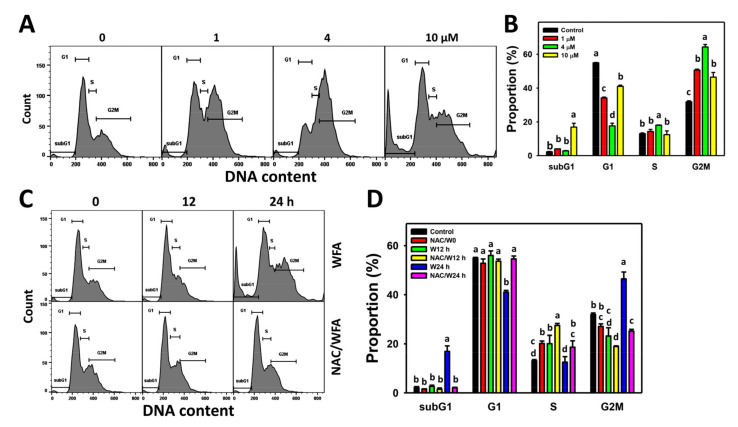
Cell cycle effect of WFA in bladder cancer cells. (**A**,**B**) Patterns and statistics for the cell cycle. Bladder cancer cells (J82) were treated with WFA (24 h, 0 to 10 μM), where the negative control for WFA (0 μM) contained 0.1% DMSO. (**C**,**D**) Pattern and statistics for NAC pretreated effects on the cell cycle of J82 cells following WFA incubation. Cells were pretreated and post treated with NAC (8 mM, 1 h) and WFA (0 and 10 μM for 12 and 24 h), respectively. They were labeled with NAC/W0, NAC/W12 h, and NAC/W24 h. Data, mean ± SD (*n* = 3). Columns showing non-overlapping lower-case letters, indicating *p* < 0.05 for multiple comparisons of the same cell cycle phase. In the example of subG1 in Figure 2D, the W24 h treatment shows “a” while others show “b”, indicating that W24 h significantly differed from others. Among non-W24 h treatments, their labeling letters are overlapping with “b”. Therefore, it shows nonsignificant differences between each other.

The profiles for the bladder cancer cell cycle following WFA incubation are shown (Figure 2A). The bladder cancer J82 cells exposed to different concentrations of WFA induced more subG1 and G2/M cells than the control (Figure 2B).

The cell cycle profiles for bladder cancer cells following NAC pre-incubation, WFA post-incubation, or both are demonstrated (Figure 2C). The bladder cancer J82 cells exposed for different incubation times of WFA induced more subG1 and G2/M cells at 24 h after WFA treatment than the control, which was inhibited by NAC pretreatment (Figure 2D).

### 3.3. WFA Triggers Annexin V-Related Apoptosis in Bladder Cancer Cells

**Figure 3:**
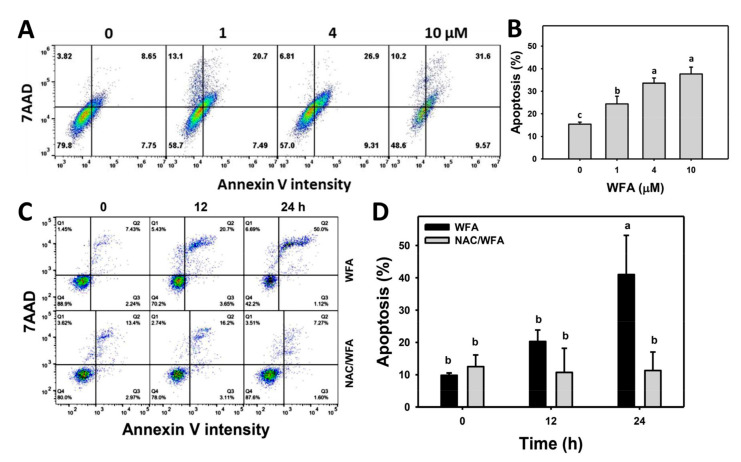
WFA induces apoptosis in bladder cancer cells. (**A**,**B**) Patterns and statistics for annexin V change. Bladder cancer cells (J82) were treated with WFA (24 h, 0 to 10 μM), where the negative control for WFA (0 μM) contained 0.1% DMSO. Annexin V (+)/7AAD (+/−) populations (%) were regarded as apoptosis (%). (**C**,**D**) Pattern and statistics for the NAC pretreated effect on annexin V expression of J82 cells were identified following WFA incubation. Cells were pretreated and post treated with NAC (8 mM, 1 h) and WFA (0 and 10 μM for 12 and 24 h), respectively. Data, mean ± SD (*n* = 3). Columns showing non-overlapping lower-case letters indicate *p* < 0.05 for multiple comparisons.

The annexin V/7AAD profiles for bladder cancer cells following WFA incubation are shown (Figure 3A). The bladder cancer J82 cells exposed to different concentrations of WFA induced more annexin V (+) cells than the control (Figure 3B).

The annexin V/7AAD profiles for bladder cancer cells following NAC pre-incubation, WFA post-incubation, or both are demonstrated (Figure 3C). The bladder cancer J82 cells exposed for different incubation times of WFA induced more annexin V (+) cells than the control, which was inhibited by NAC pretreatment (Figure 3D).

### 3.4. WFA Triggers Caspase-Related Signaling for Apoptosis in Bladder Cancer Cells 

**Figure 4:**
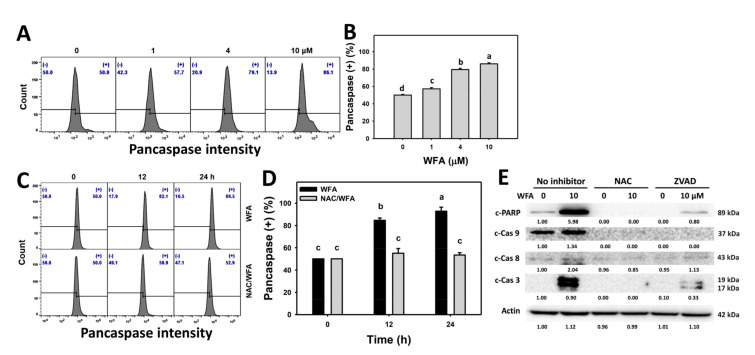
WFA activates caspases in bladder cancer cells. (**A**,**B**) Patterns and statistics for pancaspase change. Bladder cancer cells (J82) were treated with WFA (24 h, 0 to 10 μM), where the negative control for WFA (0 μM) contained 0.1% DMSO. Symbol (+) indicates pancaspase (+). (**C**,**D**) Pattern and statistics for the NAC pretreated effect on pancaspase change of J82 cells following WFA incubation. Cells were pretreated and post treated with NAC (8 mM, 1 h) and WFA (0 and 10 μM for 12 and 24 h), respectively. Data, mean ± SD (*n* = 3). Columns showing non-overlapping lower-case letters indicate *p* < 0.05 for multiple comparisons. (**E**) Western blotting analysis for NAC or ZVAD pretreated effects on apoptosis signaling expression of J82 cells following WFA incubation. Cells were pretreated and post treated with NAC (8 mM, 1 h) or apoptosis inhibitor Z-VAD-FMK (ZVAD) (20 μM, 2 h) and WFA (0 and 10 μM for 24 h), respectively, where the negative control for WFA (0 μM) contained 0.1% DMSO.

The pancaspase profiles for bladder cancer cells following WFA incubation are shown (Figure 4A). The bladder cancer J82 cells exposed to different concentrations of WFA induced more pancaspase (+) cells than the control (Figure 4B).

The pancaspase profiles for bladder cancer cells following NAC pre-incubation, WFA post-incubation, or both are demonstrated (Figure 4C). The bladder cancer J82 cells exposed for different incubation times of WFA induced more pancaspase (+) cells than the control, which was inhibited by NAC pretreatment (Figure 4D).

Since the pancaspase is nonspecific to several caspase members, such as Cas-1 and Cas-3 to -9 [21], it is important to clarify which caspases are involved in WFA-triggered apoptosis. WFA induced more c-PARP, c-Cas 9, c-Cas 8, and c-Cas 3 than the control, although the induction for c-Cas 8 was weak for bladder cancer cells (Figure 4E). The apoptosis signaling expression was suppressed by NAC and ZVAD pretreatment.

### 3.5. WFA Produces ROS and Superoxide Stresses in Bladder Cancer Cells 

**Figure 5:**
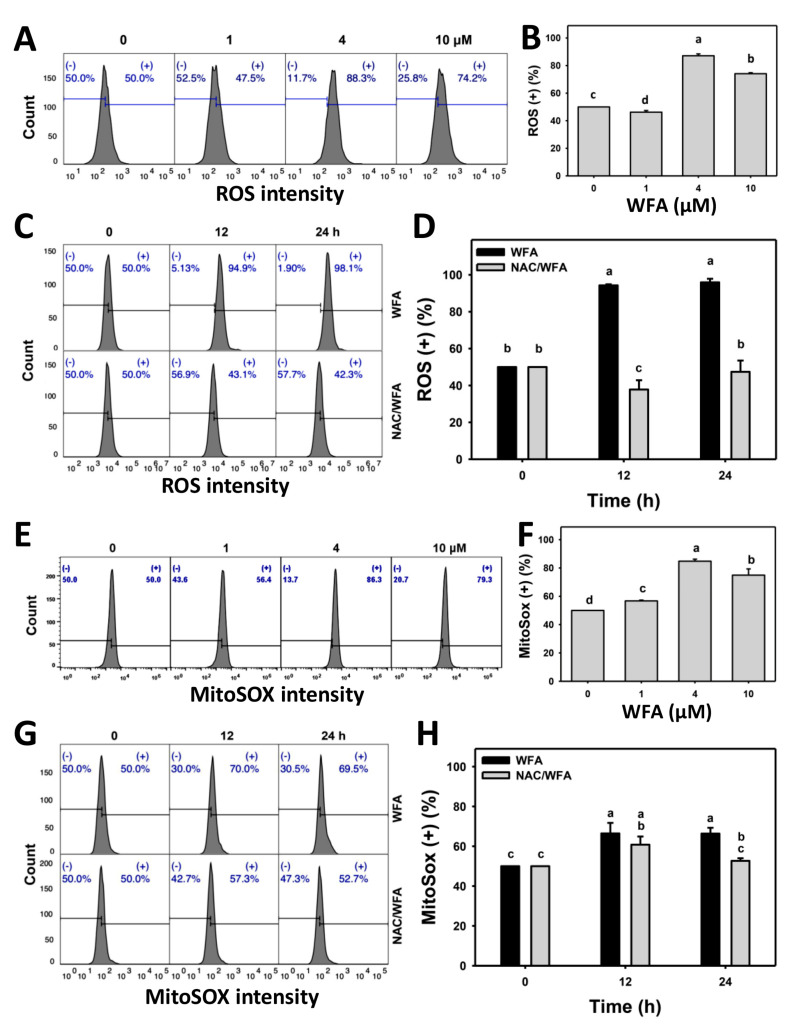
WFA induces ROS and MitoSOX generation in bladder cancer cells. (**A**,**B**,**E**,**F**) Patterns and statistics for ROS and MitoSOX changes. Bladder cancer cells (J82) were treated with WFA (24 h, 0 to 10 μM), where the negative control for WFA (0 μM) contained 0.1% DMSO. Symbol (+) indicates ROS or MitoSOX (+). (**C**,**D**,**G**,**H**) The pattern and statistics for NAC pretreated effect on ROS and MitoSOX expressions of J82 cells following WFA incubation. Cells were pretreated and post treated with NAC (8 mM, 1 h) and WFA (0 and 10 μM for 12 and 24 h), respectively. Data, mean ± SD (*n* = 3). Columns showing non-overlapping lower-case letters indicate *p* < 0.05 for multiple comparisons.

The preventive effects of NAC against antiproliferation and apoptosis as described above indicate the involvement of oxidative stress. To validate these changes of oxidative stresses, ROS and MitoSOX contents following WFA treatment were examined. The ROS and MitoSOX profiles for bladder cancer cells following WFA incubation are shown (Figure 5A,E). The bladder cancer J82 cells exposed to different concentrations of WFA induced more ROS (+) and MitoSOX (+) cells than the control (Figure 5B,F).

The ROS and MitoSOX profiles for bladder cancer cells following NAC pre-incubation, WFA post-incubation, or both are shown (Figure 5C,G). The bladder cancer J82 cells exposed for different incubation times of WFA induced more ROS (+) and MitoSOX (+) cells than the control, which was inhibited by NAC pretreatment (Figure 5D,H).

### 3.6. WFA Triggers MMP Impairment in Bladder Cancer Cells 

**Figure 6:**
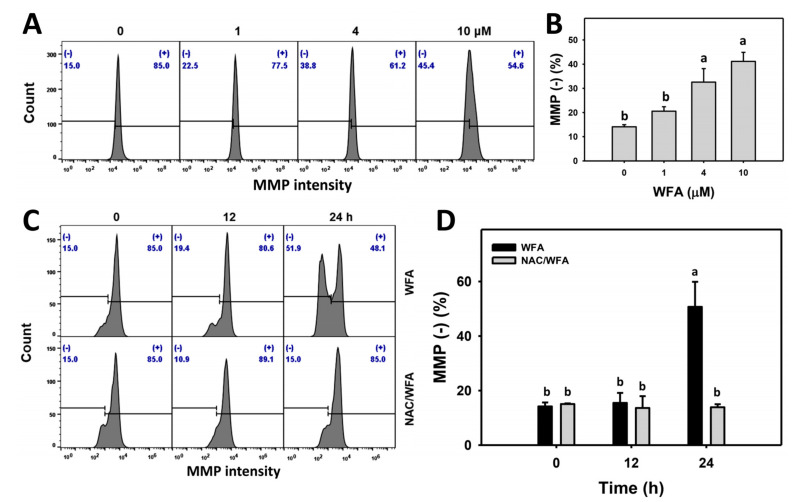
WFA induces MMP impairment in bladder cancer cells. (**A**,**B**) Patterns and statistics for MMP change. Bladder cancer cells (J82) were treated with WFA (24 h, 0 to 10 μM), where the negative control for WFA (0 μM) contained 0.1% DMSO. Symbol (-) indicates MMP (-). (**C**,**D**) The pattern and statistics for the NAC pretreated effect on MMP expression of J82 cells following WFA incubation. Cells were pretreated and post treated with NAC (8 mM, 1 h) and WFA (0 and 10 μM for 12 and 24 h), respectively. Data, mean ± SD (*n* = 3). Columns showing non-overlapping lower-case letters indicate *p* < 0.05 for multiple comparisons.

MMP also contributes to drug-induced oxidative stress, and it is essential to examine MMP contents following WFA treatment. The MMP profiles for bladder cancer cells following WFA incubation are shown (Figure 6A). The bladder cancer J82 cells exposed to different concentrations of WFA induced more MMP (-) cells than the control (Figure 6B).

The MMP profiles for bladder cancer cells following NAC pre-incubation, WFA post-incubation, or both are demonstrated (Figure 6C). The bladder cancer J82 cells exposed for different incubation times of WFA induced more MMP (-) cells than the control, which was inhibited by NAC pretreatment (Figure 6D).

### 3.7. WFA Shows Dysregulated Antioxidant Signaling in Bladder Cancer Cells

**Figure 7:**
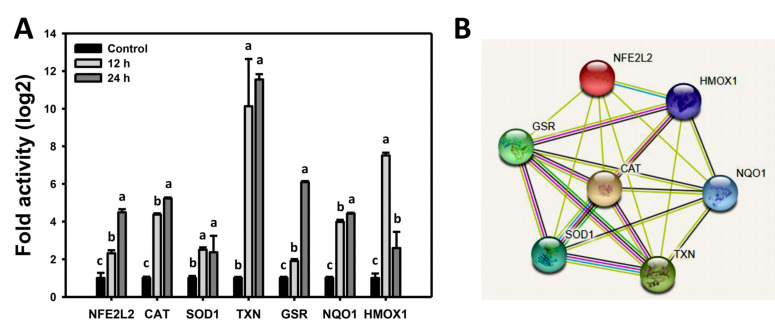
Gene expression and relationship between antioxidant signaling in WFA-treated bladder cancer cells. (**A**) Real-time PCR analysis. Cells were pretreated and post treated with NAC (8 mM, 1 h) and WFA (0 and 10 μM for 12 and 24 h), respectively. Subsequently, real-time PCR for mRNA expression in these drug-treated cells was performed. Data, mean ± SD (*n* = 3). Columns showing non-overlapping lower-case letters indicate *p* < 0.05 for multiple comparisons. (**B**) Bioinformatics analysis using STRING protein–protein interaction.

When drugs induce oxidative stress, the antioxidant gene expressions are altered [34,35]. Therefore, the involvement of the antioxidant signaling response in oxidative stress induction following WFA was further examined. WFA-induced mRNA expression for the *NFE2L2*, *CAT*, *SOD1*, *TXN*, *GSR*, *NQO1,* and *HMOX1* genes compared to the control at particular time intervals is shown (Figure 7A). The bioinformatic analysis for protein–protein interaction using STRING was conducted [36], indicating that these WFA affecting antioxidant signaling proteins are interconnected (Figure 7B).

### 3.8. WFA Triggers γH2AX and 8-OHdG DNA Damage in Bladder Cancer Cells

**Figure 8:**
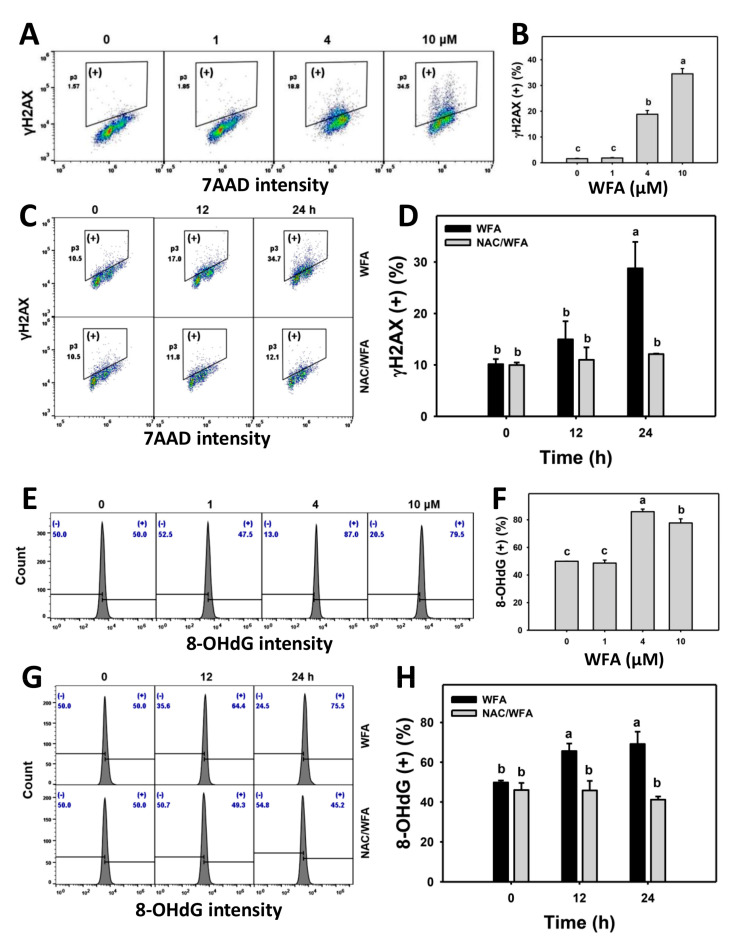
WFA induces DNA damage in bladder cancer cells. (**A**,**B**,**E**,**F**) Patterns and statistics for γH2AX and 8-OHdG changes. Bladder cancer cells (J82) were treated with WFA (24 h, 0 to 10 μM), where the negative control for WFA (0 μM) contained 0.1% DMSO. Symbol (+) indicates γH2AX or 8-OHdG (+). (**C**,**D**,**G**,**H**) Pattern and statistics for the NAC pretreated effect on the γH2AX and 8-OHdG expression of J82 cells following WFA incubation. Cells were pretreated and post treated with NAC (8 mM, 1 h) and WFA (0 and 10 μM for 12 and 24 h), respectively. Data, mean ± SD (*n* = 3). Columns showing non-overlapping lower-case letters indicate *p* < 0.05 for multiple comparisons.

γH2AX and 8-OHdG detection further evaluated the possibility that WFA induced oxidative stress, acting on DNA damage. The γH2AX and 8-OHdG profiles for bladder cancer cells following WFA incubation are shown (Figure 8A,E). The bladder cancer J82 cells exposed to different concentrations of WFA induced more γH2AX (+) and 8-OHdG (+) cells than the control (Figure 8B,F).

The γH2AX and 8-OHdG profiles for bladder cancer cells following NAC pre-incubation, WFA post-incubation, or both are demonstrated (Figure 8C,G). The bladder cancer J82 cells exposed for different incubation times of WFA induced more γH2AX (+) and 8-OHdG (+) cells than the control. This process was inhibited by NAC pretreatment (Figure 8D,H).

## 4. Discussion

WFA has reported anticancer effects on several cancer cells, but it has rarely been investigated in bladder cancer cells. The current study confirms the anticancer effect of WFA in bladder cancer cells and explores the oxidative stress mechanisms involving cell cycle arrest, apoptosis, and DNA damage. 

### 4.1. WFA Shows Differential Sensitivity to Cancer Cells

Several cancer cell lines exhibit different responses to WFA. The IC_50_ values of WFA were reported in several cancer cells, e.g., 2–3 µM (24 h MTS assay), 0.2–1.2 µM (24 h MTT assay), 1.4–9.1 µM (24 h MTT assay), 2 µM (48 h MTS assay), 0.5–1.5 µM (48 h sulforhodamine B assay), and 1–2 µM (48 h Alamar Blue assay) for oral [12], cervical [13], glioblastoma [14], pancreatic [15], lung [16], and melanoma [17] types. Under the same concentration ranges of WFA to cancer cell lines, the cell viability for normal human oral fibroblasts HGF-1 [12] and normal human lung fibroblasts MRC-5 and WI-38 [17] is higher than in cancer cells. Moreover, WFA has shown good drug safety in phase I and II clinical trials for advanced-stage osteosarcoma patients [37]. In the present study, the WFA in bladder cancer J82 cells showed an IC_50_ value of 3.1 µM after a 24 h MTS assay. Therefore, we firstly demonstrate that WFA has an antiproliferative effect on bladder cancer cells. Although WFA shows drug safety to some non-bladder normal cell lines and patients for clinical trials as mentioned above [12,17], a limitation of the present study is the experiment design lacking a non-cancer bladder cell line as a control. The selectivity of WFA in bladder cancer treatment warrants detailed investigations in the future.

### 4.2. WFA Generates Oxidative Stress on Bladder Cancer Cells

Anticancer drugs may inhibit proliferation through comprehensive oxidative stress induction. For example, a natural marine product manoalide induces cellular and mitochondrial oxidative stress in oral cancer cells [38]. Similarly, WFA generates ROS and MitoSOX in colon, oral, and breast cancer cells [12,39,40]. In addition to ROS and MitoSOX, WFA induces MMP impairment in bladder cancer cells (Figure 5 and Figure 6), indicating that this oxidative stress-inducing ability of WFA is also available to bladder cancer cells.

Mitochondria are the central system for oxidative stress generation. When mitochondria are dysfunctional, the gene expression for antioxidant genes is altered [41]. When oxidative stress dramatically accumulates, RELA activates enzymatic antioxidants, such as CAT [34]. *HMOX1* and *SOD1* mRNA is overexpressed in response to UVC/*Nepenthes* extract-induced ROS generation [35]. *GSR* mRNA expression is upregulated by physapruin A-induced ROS generation in breast cancer cells [42]. High oxidative stress in cultured oocytes results in overexpression of mRNA for *CAT, SOD1*, *SOD2,* and *GSR* antioxidant genes [43]. Similarly, mRNA and protein levels for *CAT*, *SOD1*, and *HMOX1* genes were activated in UVC-irradiated mice [44]. UVC was also reported to induce ROS generation in oral cancer cells [35,45]. Oxidative stress also activates TXN, which is a target of NFE2L2 [46]. *NQO1* knockdown inhibits ROS generation in prostate cancer cells [47]; therefore, *NQO1* upregulation may be associated with ROS induction. 

Similarly, WFA upregulates the mRNA expression of antioxidant genes (*NFE2L2*, *CAT*, *SOD1*, *TXN*, *GSR*, *NQO1,* and *HMOX1*) (Figure 7) in bladder cancer cells associated with oxidative stress generation (Figure 5 and Figure 6). Therefore, these results suggest that this antioxidant mRNA expression is upregulated in response to WFA-generated oxidative stress. Still, mRNA expression fails to overcome the high level of oxidative stress. 

There are three SOD isoforms, including cytoplasmic (SOD1), mitochondrial (SOD2), and extracellular (SOD3) types, in mammals [48]. The *SOD1* mRNA was activated; however, the expression of *SOD2* and *SOD3* genes was not examined in the present study. Since WFA induces MitoSOX generation in oral cancer cells, mitochondrial SOD2 expression may play a vital role in WFA-induced oxidative stress. 

Moreover, WFA-activated antioxidant signaling by upregulating mRNA expression lacks protein-level confirmation for antioxidant genes. Therefore, it warrants detailed investigations of both the mRNA and protein expression of all SOD isoforms in WFA-treated oral cancer cells in the future to provide a comprehensive view of the mechanisms of action of WFA-induced oxidative stress.

Both cellular and mitochondrial oxidative stress has been demonstrated to trigger apoptosis in the present study. Lipid peroxidation was also reported as a proapoptotic factor [49]. However, the role of lipid peroxidation was not examined. Selective anticancer cytotoxicity may be caused by lipid peroxidation [50]. Further, 4-hydroxynonenal (4-HNE) [51,52] or 4-hydroxy-2-nonenal (HNE) [53] is an α,β-unsaturated hydroxyalkenal generated by lipid peroxidation. For example, polyunsaturated fatty acids (PUFA) can generate the peroxidation product HNE or 4-HNE, which is known to act as a second messenger of ROS [52,54], the signaling molecule regulating cell growth. The potential anticancer effects of HNE can be attenuated by GSH and NAC [55]. It warrants further detailed investigation that explores the role of lipid peroxidation in WFA-induced oxidative stress and apoptosis as well as selective killing of bladder cancer cells.

### 4.3. WFA Drives Apoptosis and Causes DNA Damage of Bladder Cancer Cells

Oxidative stress-regulating drugs may modulate the effects of apoptosis or DNA damage for antiproliferation of cancer cells [6]. A number of reports indicate that WFA triggers apoptosis in several types of cancer cells [14,17,56,57]. WFA induces more intrinsic apoptotic c-Cas 9 expression in glioblastoma cell studies than extrinsic apoptotic c-Cas 8 expression [14], which has the same tendency for bladder cancer cells (Figure 4). In the oral cancer cell study, c-Cas 8 and c-Cas 9 were induced in the WFA concentration at 60% and 70% viability but declined at a concentration of 50% viability [12]. Therefore, the induction of intrinsic and extrinsic apoptosis signaling may depend on the context of cancer cell types.

Except for oral [12] and breast [58] cancer cells, the finding that WFA induces DNA damage is rarely reported in other cancer cells. WFA overexpresses γH2AX in breast [58], oral [12], and bladder (Figure 8) cancer cells. Moreover, WFA-induced oxidative stress further attacks DNA to generate oxidative DNA damage, as shown in oral [12] and bladder (Figure 8) cancer cells.

### 4.4. WFA Blocks G2/M Progression on Bladder Cancer Cells

WFA arrests G2/M progression in several types of cancer cells, such as gastric [59], osteosarcoma [60], leukemia [61], breast [62], oral [12], colon [39], and glioblastoma [14] cancer cells. Furthermore, prostate cancer cells following WFA treatment induce a mitotic catastrophe [63]. Consistently, WFA shows higher G2/M populations in bladder cancer cells (Figure 2).

### 4.5. NAC Suppresses Antiproliferation Mechanisms of WFA on Bladder Cancer Cells

The ROS inhibitor (NAC) pretreatment confirmed the function of oxidative stress in several WFA anticancer cell studies. For example, NAC suppresses WFA-induced apoptosis in head and neck cancer AMC-HN4 cells [64]. In addition, NAC alleviates WFA-mediated endoplasmic reticulum stress and apoptosis in renal cancer cells [65]. NAC also suppresses ROS generation, MMP destruction, and apoptosis in colon cancer cells [39]. Similarly, NAC suppresses WFA-induced changes in bladder cancer cells, including antiproliferation, G2/M arrest, apoptosis expression for annexin V, pancaspase function, caspase signaling protein expression, ROS induction, MMP destruction, double-strand breaks, and oxidative DNA damage. These findings suggest that oxidative stress regulates the antiproliferation effect and mechanism in bladder cancer cells following WFA treatment. The NAC effect on the mRNA and protein expression for antioxidant signaling was not examined in the present study. Thus, the relationship between antioxidant signaling and oxidative stress is still unclear. More detailed investigations of NAC pretreatment effects on antioxidant signaling are warranted in the future.

### 4.6. Potential Targets of WFA

Heat shock protein 90 (HSP90) [66] and annexin II [67] were reported as additional targets of WFA. For example, WFA binds to HSP90 to inhibit HSP90 chaperone activity [66] and reduces the interaction between the FA complementation group A (FANCA) and HSP90 to reduce the single-strand annealing sub-pathway (SSA) repair, leading to double-strand break (DSB) accumulation [68] and apoptosis. This finding can support our results that WFA induces DSB and apoptosis. Moreover, WFA is reported to covalently bind to annexin II to change the cytoskeleton network and inhibit cancer cell migration and invasion [67], which was not addressed in the present study.

## 5. Conclusions

The antiproliferation effect of WFA treatment on bladder cancer cells has been little studied as yet. Here, we provided the first evidence that WFA exhibits an antiproliferation-modulating impact on bladder cancer cells in an oxidative stress-dependent manner. Antiproliferation generally increased with WFA doses and exposure time. Mechanistically, WFA generates cellular and mitochondrial oxidative stresses with the destruction of bladder cancer cells caused by the increase of ROS/MitoSOX and the decrease of MMP. Furthermore, WFA-induced oxidative stress was associated with the upregulation of antioxidant signaling expressions. Moreover, NAC pretreatment reverted oxidative stresses and their associated responses to G2/M arrest, apoptosis, and DNA damage in bladder cancer cells. Therefore, WFA causes oxidative stress-dependent antiproliferation and apoptosis effects in bladder cancer cells.

## Data Availability

Data is contained within the article.
